# Comprehensive Profiling and Quantification of Ginsenosides in the Root, Stem, Leaf, and Berry of *Panax ginseng* by UPLC-QTOF/MS

**DOI:** 10.3390/molecules22122147

**Published:** 2017-12-04

**Authors:** Jae Won Lee, Bo-Ram Choi, Young-Chang Kim, Doo Jin Choi, Young-Seob Lee, Geum-Soog Kim, Nam-In Baek, Seung-Yu Kim, Dae Young Lee

**Affiliations:** 1 Department of Herbal Crop Research, National Institute of Horticultural and Herbal Science, RDA, Eumseong 27709, Korea; jaewon3@gmail.com (J.W.L.); bmcbr@naver.com (B.-R.C.); ycpiano@rda.go.kr (Y.-C.K.); cdj0105@korea.kr (D.J.C.); youngseoblee@korea.kr (Y.-S.L.); kimgs@rda.go.kr (G.-S.K.); 2 Department of Oriental Medicine Biotechnology, Kyung Hee University, Yongin 17104, Korea; nibaek@khu.ac.kr; 3 Protected Horticulture Research Institute, National Institute of Horticultural and Herbal Science, RDA, Haman 52054, Korea; kimsy@rda.go.kr

**Keywords:** *Panax ginseng*, ginsenosides, UPLC-QTOF/MS

## Abstract

The effective production and usage of ginsenosides, given their distinct pharmacological effects, are receiving increasing amounts of attention. As the ginsenosides content differs in different parts of *Panax ginseng*, we wanted to assess and compare the ginsenosides content in the ginseng roots, leave, stems, and berries. To extract the ginsenosides, 70% (*v/v*) methanol was used. The optimal ultra-performance liquid chromatography-quadrupole time of flight mass spectrometry (UPLC-QTOF/MS) method was used to profile various ginsenosides from the different parts of *P. ginseng*. The datasets were then subjected to multivariate analysis including principal component analysis (PCA) and hierarchical clustering analysis (HCA). A UPLC-QTOF/MS method with an in-house library was constructed to profile 58 ginsenosides. With this method, a total of 39 ginsenosides were successfully identified and quantified in the ginseng roots, leave, stem, and berries. PCA and HCA characterized the different ginsenosides compositions from the different parts. The quantitative ginsenoside contents were also characterized from each plant part. The results of this study indicate that the UPLC-QTOF/MS method can be an effective tool to characterize various ginsenosides from the different parts of *P. ginseng*.

## 1. Introduction

In the herbal markets and food industries of Korea and other East Asian countries, *Panax ginseng C.A. Meyer* is one of the most important herbal plants. Its roots have been used as a main constituent in oriental medicine [1,2]. *P. ginseng* root contains many bioactive compounds including ginsenosides, peptides, polyacetylenic alcohols, fatty acids, and essential oils [3]. Among these compounds, ginsenosides are considered critical ingredients, having various pharmacological effects, such as enhancing the immune system, and anti-tumor and anti-diabetes activities [4,5,6,7]. Each ginsenoside has specific activities, and the efficacy of ginseng is determined by the ginsenoside contents. Due to its biological utility, the production and use of ginsenosides has been receiving increased attention. More than 150 ginsenosides have been isolated and identified from the *P. ginseng* plant [8,9]. In particular, its distinct parts including the roots, leaves, stems, and berries are considered as good sources of ginsenosides [10,11,12]. The qualitative and quantitative ginsenoside contents may differ depending on the plant part of *P. ginseng* [13,14]. Hence, to evaluate the efficacy of different ginseng parts and their effective production of ginsenosides, profiling the various ginsenosides from the distinct parts of *P. ginseng* is required.

To assess the levels of various ginsenosides from a complex *P. ginseng* extract, a sensitive and reliable analytical method is necessary. To analyze ginsenosides, high performance liquid chromatography (HPLC) coupled with a UV detector [15,16], or an evaporative light scattering detector (ELSD) [17,18] are typically used. However, due to the limited availability of standard samples, only a few ginsenosides have been quantified. Liquid chromatography (LC) coupled with electrospray ionization/mass spectrometry (ESI/MS) has emerged as a robust tool for the comprehensive profiling of various ginsenosides [19,20,21]. Within MS, a full mass scan based on quadrupole time of flight (QTOF)/MS is a sensitive detector used to measure the exact mass values of target and non-target compounds [22,23]. The ultra-performance LC (UPLC) system provides high resolution separation using a small particle size column, and is effective for analyzing various compounds in a complex mixture [24].

In this study, UPLC-QTOF/MS with an in-house library was used to perform a comprehensive profiling of ginsenosides from the roots, leave, stems, and berries of *P. ginseng*. To construct an in-house library and the quantification of ginsenosides, we used a total of 58 standard samples, including malonyl-ginsenosides (MGRs), isolated in our laboratory. The qualitative and quantitative contents of ginsenosides were characterized in the distinct parts of *P. ginseng*.

## 2. Materials and Methods 

### 2.1. Standard Constituents and Reagents

HPLC-grade methanol (MeOH), water, and ACN were obtained from Fisher Scientific Korea (Seoul, Korea). HPLC-grade formic acid was purchased from Fluka Analytical, Sigma Aldrich Chemie GmbH (Steinheim, Germany). The ginsenoside standards used in this study were isolated and purified from *P. ginseng* roots and red ginseng by a series of chromatography procedures in our laboratory, and the information for 30 ginsenosides was reported in our previous study [25]. The structures of other ginsenosides were determined based on the spectroscopic data (MS, hydrogen nuclear magnetic resonance (^1^H-NMR), and carbon-13 (^13^C)-NMR) in the previous literatures: fKa [26], nR4 [27], nR2-R [28], Rh1-R [29], Ra3 [26], M-Rb1 [30], M-Rc [31], M-Rb2 [30], M-Rd [31], CO [32], GyL [33], GyLI [33], nFt1 [34], Rg3-R [29], Mc [35], and CY [35]. Rf, Rk1, Ro, Rg2-R, PPD, and PPT were purchased from Sigma Aldrich (St. Louis, MO, USA). Rk3, Rh4, GyXVII, and XLIX were purchased from Chemfaces (Wuhan, China). Rk2 and GyA were purchased from ALB technology (Mongkok Kowloon, Hong Kong, China).

### 2.2. Panax ginseng Samples 

*Panax ginseng* was cultivated in the experimental field of the National Institute of Horticultural and Herbal Science, Rural Development Administration (RDA) at Chungbuk, Eumseong, Korea (36° N, 127° E), according to the “ginseng GAP standard cultivation guide” protocol developed by RDA, Republic of Korea [36]. A voucher specimen (NIHHS-14S01) was deposited at the Herbarium of the Department of Herbal Crop Research, National Institute of Horticultural and Herbal Science, Rural Development Administration, Eumseong, Republic of Korea. Six years into its growing season, in the month of July, the roots, stems and leaves, and berries were harvested, and stored in a deep freezer at −80 °C until further processing.

### 2.3. Extraction of Ginsenosides

The samples of ginseng roots, stems and leaves, and berries were dried at 40 °C in a forced-air, convection-drying oven for 48 h after washing, and then weighed. For the ginseng root, the main roots were used for experiments after removing the lateral and fine roots. Each sample was ground (<0.5 mm) using a mixer (Hanil, Seoul, Korea) and thoroughly mixed, and subsamples were homogenized further using a Retsch MM400 mixer mill (Retsch GmbH, Haan, Germany) for analyses. Fine powder was weighed (200 mg), suspended in 2 mL of 70% (*v*/*v*) methanol, and ultrasonically extracted for 30 min at 50 °C. Each sample was centrifuged at 13,500 rpm for 5 min. The solution was filtered through a syringe filter (0.22 µm) and injected directly into the UPLC system.

### 2.4. UPLC-QTOF/MS Conditions for Ginsenoside Profiling

UPLC was performed using a Waters ACQUITY H-Class UPLC (Waters Corp., Milford, MA, USA) with an ACQUITY BEH C18 column (2.1 mm × 100 mm; 1.7 µm). The column oven was maintained at 40 °C, and the mobile phases included solvent A [water and 0.1% formic acid (*v*/*v*)] and solvent B [acetonitrile and 0.1% formic acid (*v*/*v*)]. The elution gradient was as follows: 0–0.5 min, B 15%; 0.5–1 min, B 15–20%; 1–6 min, B 20%; 6–13 min, B 20–30%; 13–23 min, B 30–35%; 23–24 min, B 35–38%; 24–27 min, B 38–60%; 27–31 min, B 60–90%; 31–32 min, B 90–15%; and 32–35 min, B 15%. The flow rate was 450 μL/min, and the injection volume was 2 μL for each run.

Next, MS analysis was performed using a Waters Xevo G2-S QTOF MS (Waters Corp., Milford, MA, USA) operating in negative ion mode. The mass spectrometers performed alternating high- and low-energy scans, known as the MS^E^ acquisition mode. The operating parameters were set as follows: cone voltage 40 V; capillary 3.0 kV; source temperature 120 °C; desolvation temperature 550 °C; cone gas flow 30 L/h; and desolvation gas flow at 800 L/h. Accurate mass measurements were obtained by means of an automated calibration delivery system that contained an internal reference [Leucine-enkephalin, *m*/*z* 554.262 (ESI-)]. Data were collected between 100 and 2000 *m*/*z*.

### 2.5. Data Processing and Multivariate Analysis

All MS^E^ data were collected and processed using UNIFI 1.8 (Waters Corp., Milford, MA, USA). Data within UNIFI 1.8 were passed through the apex peak detection and alignment processing algorithms. The intensity of each ion was normalized with respect to the total ion count to generate a data matrix having RT, *m*/*z* value, and the normalized peak area. Charged species, salt adducts, and fragments were all automatically aligned and grouped. The three-dimensional data, including peak number (RT-*m*/*z* pair), sample name, and normalized peak areas, were exported to the EZinfo software 3.0.3 (UMETRICS) for PCA. The processed data were also entered into the MetaboAnalyst website (http://www.metaboanalyst.ca) to perform HCA.

## 3. Results and Discussion

### 3.1. UPLC-QTOF/MS with an In-House Library to Profile Various Ginsenosides

The sensitivity of MS-based analytical methods has increased to enable the detection of compounds with limited abundance in biological samples. Thus, LC-MS methods have been widely used to analyze major compounds in various herbal medicines. For the detailed characterization of *P. ginseng*, assessing the levels of both the high- and low-abundance ginsenosides is important. In our previous study [25], UPLC-QTOF/MS was used to analyze 30 ginsenosides. The optimal UPLC method was effective for separating various ginsenosides. In the identification of ginsenosides, QTOF/MS was also sensitive and reliable for measuring the exact mass values of the compounds. In this study, the LC-MS method was simultaneously applied to profile 58 ginsenosides. Figure S1 represented the molecular structures of these ginsenosides. First, these ginsenoside standards were purchased or obtained from the experiments by isolating and purifying them from *P. ginseng*. In our laboratory, we collected four MGRs that contained a malonyl residue, M-Rb1, M-Rb2, M-Rc, and M-Rd, which is linked to the glucose in the compounds. Isolating and purifying the MGRs from the extracted mixture of *P. ginseng* is difficult in practice. Due to their instability when exposed to heat, care must be taken when handling and preserving MGR compounds. 

**Figure 1:**
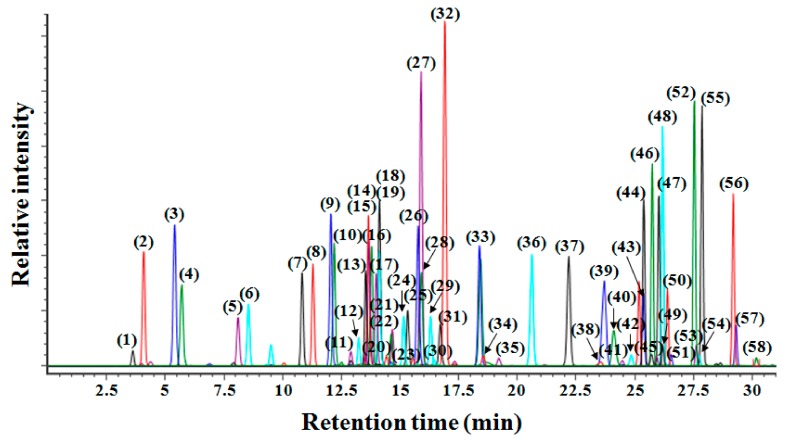
Base peak intensity (BPI) chromatogram of 58 ginsenoside standards. The peaks of (**1**)–(**58**) are aligned with the 58 ginsenosides listed in Table 1.

Next, for the comprehensive profiling of various ginsenosides, 58 ginsenoside standards were used to optimize the UPLC-QTOF/MS conditions. By using the UPLC system with an ACQUITY BEH C18 column, these standards were well separated after 30 min at a flow rate of 450 µL/min. The base peak intensity (BPI) chromatograms of the 58 ginsenosides are shown in Figure 1. Among these ginsenosides, 16 isomeric well-separated pairs were found: C_36_H_60_O_7_ (Rk2 or Rh3), C_36_H_60_O_8_ (Rk3, Rh4, fKa, or Rh6), C_36_H_62_O_8_ (Rh2 or CK), C_36_H_62_O_9_ (Rh1, Rh1-R, or F1), C_41_H_70_O_12_ (Mc or CY), C_41_H_70_O_13_ (nR2, F5, nR2-R, or F3), C_42_H_70_O_12_ (Rg4, F4, Rk1, or Rg5), C_42_H_72_O_13_ (Rg2, Rg2-R, F2, Rg3, or Rg3-R), C_42_H_72_O_14_ (Rg1, pF11, Rf, GyL, or GyLI), C_47_H_80_O_17_ (nFe, CO, or nFt1), C_48_H_82_O_18_ (Re, Rd, or GyXVII), C_48_H_82_O_19_ (gRf or vR4), C_53_H_90_O_22_ (Rc, Rb2, or Rb3), C_56_H_92_O_25_ (M-Rc or M-Rb2), C_58_H_98_O_26_ (Ra1 or Ra2), and C_59_H_100_O_27_ (nR4 or Ra3). Although several peaks overlapped with each other, identifying each peak was possible, based on its exact mass values obtained by QTOF/MS. In the negative ion mode, ginsenosides were detected as [M + COOH]^−^ ions and [M − H]^−^ ions with high mass accuracy (<2.5 ppm). For the exact identification of ginsenosides, we constructed an in-house library using the UNIFI software. Information about the compound’s name, molecular formula, retention time (RT), mass accuracy, and adduct was listed in the library for the 58 ginsenosides (Table 1). Next, a validation study was performed to prove the performance of the UPLC-QTOF/MS method for profiling the ginsenosides. For this, the standard curves of the 58 ginsenosides were constructed, and their linearity range and correlation (*R*^2^) were calculated. The limits of detection (LODs) of each standard are provided in Table S1. The *R*^2^ of each ginsenoside analysis was, at minimum, above 0.9908, proving the high reliability of the quantitative analysis of the ginsenosides. LODs also showed the high sensitivity of this method for detecting ginsenosides. For example, in the case of Ra3, Ro, M-Rb2, Rd, GyL, GyLI, Rg3, Mc, and Cy, the two picogram (pg) of these compounds was detected by UPLC-QTOF/MS.

### 3.2. Profiling of Various Ginsenosides from the Roots, Stems, Leaves, and Berries of Panax ginseng

**Figure 2:**
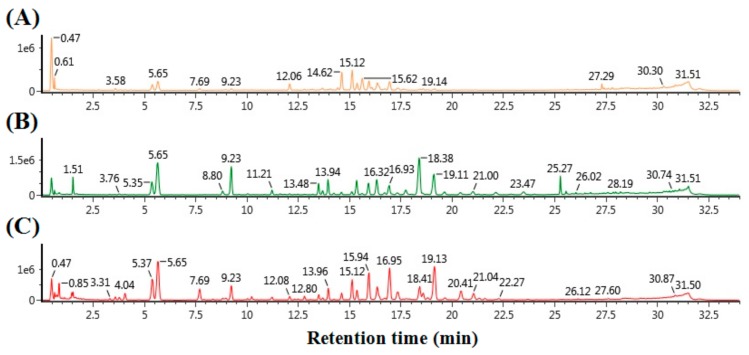
BPI chromatogram of ginsenoside extracts from three samples of (**A**) ginseng roots (GR), (**B**) stems and leaves (GS), and (**C**) berries (GB).

In this study, the constructed UPLC-QTOF/MS with an in-house library was applied to profile various ginsenosides from the roots, stems, leaves, and berries of *P. ginseng*. The numbers of individual samples were as follows: ginseng root (GR) (*n* = 10), stem and leaf (GS) (*n* = 10), and berry (GB) (*n* = 10). To extract various ginsenosides from individual samples, 70% (*v*/*v*) methanol was used. The extracts were analyzed using UPLC-QTOF/MS; each piece of data was then processed using the UNIFI software. Figure 2 shows the representative BPI chromatograms of ginsenoside extracts from GR, GS, and GB. UPLC was able to separate the ginsenosides and other metabolites in 30 min. The intensities of several peaks were different dependent on the sample. For example, overall, the GR sample had lower intensity peaks compared to the other samples. The GS sample had higher intensity peaks than the GB sample, eluted for 5–10 min and 18–26 min. However, the GB sample had higher intensity peaks than the GS sample, eluted for 5–10 min. These demonstrated that GR, GS, and GB have distinct metabolite compositions, including ginsenosides.

**Figure 3:**
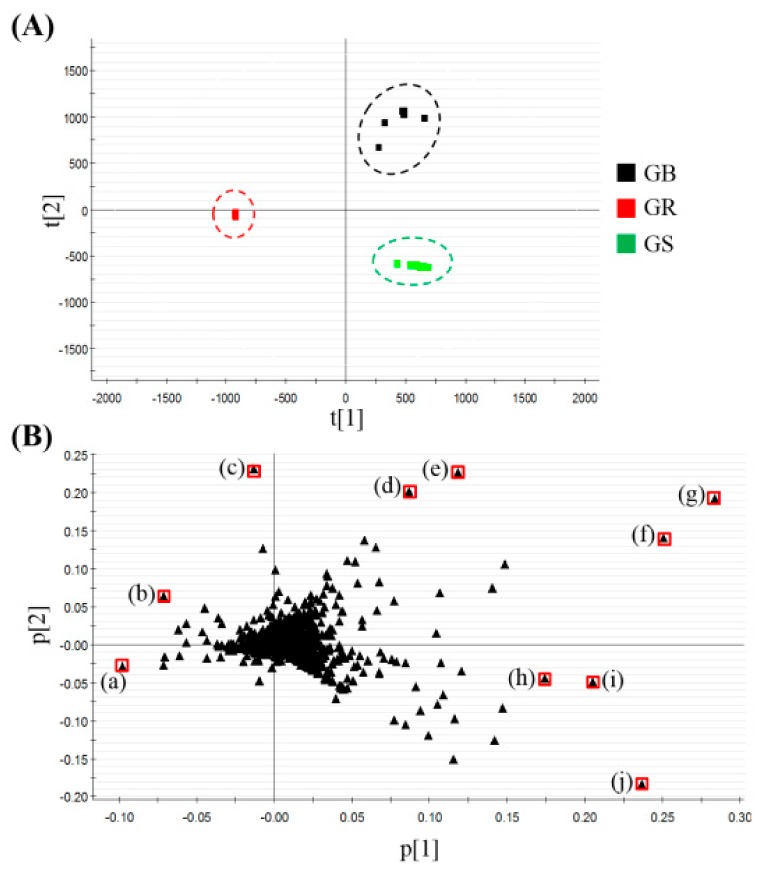
(**A**) Principal component analysis (PCA) score plot and (**B**) loading plot of ginsenoside extracts from ginseng roots (GR) (*n* = 10), stems and leaves (GS) (*n* = 10), and berries (GB) (*n* = 10). In the PCA loading plot, each point was identified as follows: ginsenoside (a) Ro (b) Rb1, (c) M-Rb1, (d) M-Rc, (e) M-Rb2, (f) M-Rd, (g) Re, (h) F3, (i) unknown, and (j) Rd.

**Figure 4:**
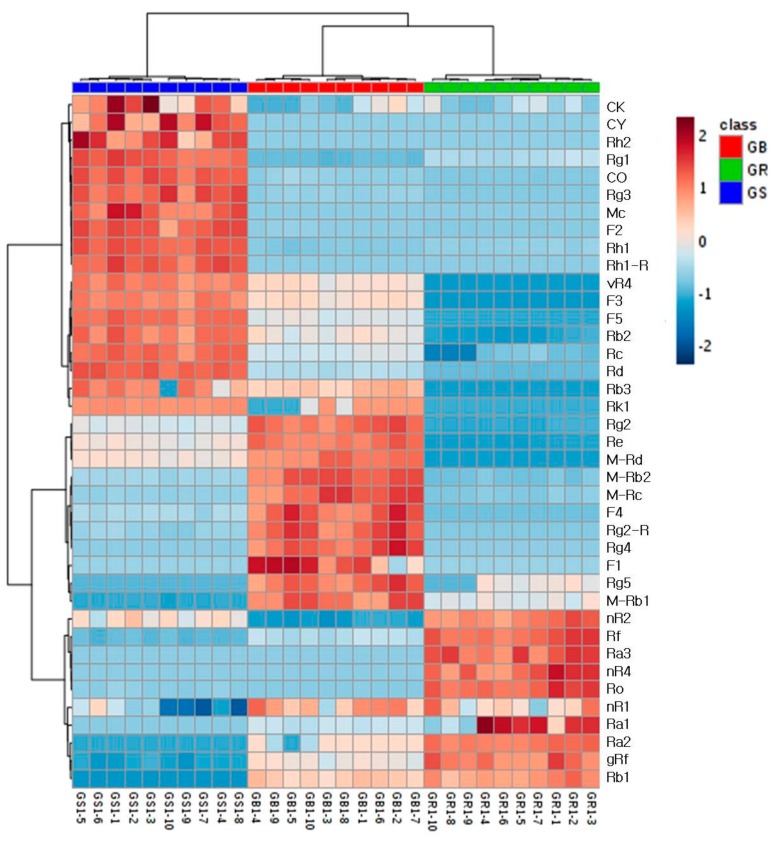
Hierarchical clustering analysis of the 39 ginsenoside datasets from the ginseng root (GR) (*n* = 10), stems and leaves (GS) (*n* = 10), and berries (GB) (*n* =10).

First, all the processed data were exported to the EZinfo software for principal component analysis (PCA). PCA helps visualize the general clustering trends among individual samples, and the scatter of the points indicates the similarities or differences between the metabolite data of the samples. In the score plot, the GR, GS, and GB were well-differentiated (Figure 3A). Moreover, in the loading plot, we identified the main variables that differentiate the three groups in the score plot. Each point represented the *m*/*z*-RT pairs of molecules. Based on the in-house library, the nine variables of the loading plot were identified as several ginsenosides (Figure 3B). Next, in the data processing, a total of 39 ginsenosides were identified from the three samples. Using the in-house library, the numbers of identified ginsenosides from three samples were as follows: 27 in GR, 32 in GS, and 29 in GB. The ginsenosides datasets were then subjected to hierarchical clustering analysis (HCA). As a result, the three groups were clearly differentiated. The different levels of the 39 ginsenosides in the ginseng roots, stems and leave, and berries are described in the heatmap in Figure 4. This demonstrated the major ginsenosides that were found in each ginseng part.

### 3.3. Quantification of Ginsenosides from the Roots, Stems and Leaves, and Berries of Panax ginseng

Next, we quantified the 39 ginsenosides in the individual samples. The standard curve of each ginsenoside was applied to determine its actual amount in the ginseng samples. The quantification was based on 10 biological replicate analysis of each ginseng sample (*n* = 10), and the amounts are described as mean ± SD in Table 2. This displays the qualitative and quantitative ginsenoside contents in the different *P. ginseng* parts. Previously, Wang et al. has reported the HPLC-UV–based quantification of 12 ginsenosides from ginseng root, rootlet, leaf, and berry [14]. From the five parts such as root, root-hair, rhizome, stem, and leaf, Shi et al. quantified seven ginsenosides by HPLC-UV [13], and Qu et al. quantified 12 ginsenosides by HPLC-ELSD [36]. Compared to these reports, this study detail characterized a total of 39 ginsenosides with high- or low-abundance from ginseng root, stem and leaf, and berry. As our results, ginsenosides, such as nR4, Ra2, Ra3, and Ra1, were uniquely found in GR. Furthermore, GS uniquely contained Rb3, Mc, CY, Rh2, and Rh1-R. F1 and Rg4 were only detected in GB. Although most of unique ginsenosides are low-abundant, their profiles are valuable to characterize the ginsenoside contents of each ginseng part. In the quantification, GR had highest amounts of Rf, Rb1, and Ro, compared to the other samples. GS had highest amounts of Rg1, vR4, F5, Rh1, F3, Rc, Rb2, Rd, CO, F2, Rg3, CK, and Rk1. GB had highest amounts of gRf, nR1, Re, nR2, Rg2, Rg2-R, M-Rb1, M-Rc, M-Rb2, M-Rd, F4, and Rg5. Previously, two papers have reported that GR had the highest amounts of Rb1, GB had the highest amounts of Rg2, and GS had the highest amounts of Rg1, Rh1, Rd, and Rg3 [14,36]. Interestingly, these results have been matched to ours. Rb3 and Rh1-R are also equally analyzed from GS [13,14]. On the other hand, Wang et al. has reported that Re and Rc are mainly consisted in GS and GR, respectively [14]. As our results, GB had highest amounts of Re, and this is matched to another previous paper [37]. The quantification of several ginsenosides can differ depending on ginseng’s cultivars and growing conditions. 

Each ginsenoside has specific activities, and the efficacies of GR, GS, and GB are determined by the ginsenoside contents. Here, we focused on the pharmacological activities of ginsenosides which are mainly or uniquely consisted in each ginseng part. By searching previous literatures [5,6,15,38,39,40,41,42,43,44,45,46], we found six kinds of activities in 17 ginsenosides (Table S2). In summary, three ginsenosides (Rf, Rb1, Ro) consisted in GR shows anti-inflammation effect and lymphocyte proliferative capacity. Moreover, 10 ginsenosides (Rg1, Rc, Rb2, Rd, F2, Rg3, CK, Rb3, Rh2, Rh1-R) consisted in GS shows six activities (anti-cancer, anti-diabetes, anti-inflammation, lymphocyte proliferative capacity, immunomodulating activity, chemopreventive effect). Lastly, four ginsenosides (Re, Rg2, Rg2-R, F1) consisted in GB shows four activities (anti-cancer, anti-diabetes, anti-inflammation, lymphocyte proliferative capacity). These results demonstrated that not only GR, but also GS and GB are good sources to obtain ginsenosides having pharmacological activities. By detail profiling of various ginsenosides, it was possible to find the efficacies of ginseng root, stems, leaves, and berries.

## 4. Conclusions

In conclusion, this study optimized an analytical method based on UPLC-QTOF/MS with an in-house library to profile 58 ginsenosides. Furthermore, using this method, we determined the quantitative contents of 39 ginsenosides from GR, GS, and GB. These results demonstrated that not only the ginseng root, but also the stems, leaves, and berries are good sources of ginsenosides. Each ginsenoside has specific activities. Thus, in the basis of ginsenosides profiling and quantification, we can assess the pharmacological utility of different ginseng parts. Moreover, ginsenoside contents may differ depending on ginseng’s cultivars, growing years, growing regions, etc. Finally, the proposed UPLC-QTOF/MS method for ginsenoside profiling is a good tool for evaluating various ginseng samples as sources for ginsenosides. 

## Figures and Tables

**Table 1:** In-house library for the analysis of 58 ginsenosides using ultra-performance liquid chromatography-quadrupole time of flight mass spectrometry (UPLC-QTOF/MS).

No.	RT (min)	Ginsenosides	Molecular Formula	Expected Neutral Mass (Da)	Observed Neutral Mass (Da)	QTOF/MS (ESI-) (*m*/*z*)	Mass Accuracy (ppm)	Adducts
**1**	3.58	20-O-Glucoginsenoside Rf (gRf)	C_48_H_82_O_19_	962.545	962.5453	1007.5435	0.26	+HCOO
**2**	4.07	Notoginsenoside R1 (nR1)	C_47_H_80_O_18_	932.5345	932.5343	977.5325	0.2	+HCOO
**3**	5.36	Ginsenoisde Rg1 (Rg1)	C_42_H_72_O_14_	800.4922	800.4908	845.489	1.62	+HCOO
**4**	5.66	Ginsenoside Re (Re)	C_48_H_82_O_18_	946.5501	946.5503	991.5485	0.16	+HCOO
**5**	8.05	Floralginsenoside Ka (fKa)	C_36_H_62_O_11_	670.4292	670.4295	715.4277	0.34	+HCOO
**6**	8.46	Ginsenoside Rh6 (Rh6)	C_36_H_62_O_11_	670.4292	670.4291	715.4273	0.19	+HCOO
**7**	10.72	Ginsenoside Rh23 (Rh23)	C_37_H_64_O_10_	668.4499	668.4507	713.4489	1.06	+HCOO
**8**	11.2	Vinaginsenoside R4 (vR4)	C_48_H_82_O_19_	962.545	962.5452	1007.5434	0.19	+HCOO
**9**	11.94	Pseudo-ginsenoside F11 (pF11)	C_42_H_72_O_14_	800.4922	800.4932	845.4914	1.22	+HCOO
**10**	12.11	Ginsenoside Rf (Rf)	C_42_H_72_O_14_	800.4922	800.4922	845.4904	0.01	+HCOO
**11**	12.81	Notoginsenoside R2 (nR2)	C_41_H_70_O_13_	770.4816	770.4823	815.4805	0.83	+HCOO
**12**	13.15	Notoginsenoside R4 (nR4)	C_59_H_100_O_27_	1240.6452	1240.6455	1285.6437	0.2	+HCOO
**13**	13.43	Ginsenoside F5 (F5)	C_41_H_70_O_13_	770.4816	770.4819	815.4801	0.33	+HCOO
**14**	13.56	Ginsenoside Rh1 (Rh1)	C_36_H_62_O_9_	638.4394	638.4392	683.4374	0.31	+HCOO
**15**	13.58	20(R)-Notoginsenoside R2 (nR2-R)	C_41_H_70_O_13_	770.4816	770.4815	815.4797	0.18	+HCOO
**16**	13.7	Ginsenoside Rg2 (Rg2)	C_42_H_72_O_13_	784.4973	784.4984	829.4966	1.38	+HCOO
**17**	13.9	Ginsenoside F3 (F3)	C_41_H_70_O_13_	770.4816	770.4811	815.4793	0.63	+HCOO
**18**	14.03	20(R)-Ginsenoside Rg2 (Rg2-R)	C_42_H_72_O_13_	784.4973	784.496	829.4942	1.58	+HCOO
**19**	14.03	20(R)-Ginsenoside Rh1 (Rh1-R)	C_36_H_62_O_9_	638.4394	638.4397	683.4379	0.47	+HCOO
**20**	14.35	Ginsenoside Ra2 (Ra2)	C_58_H_98_O_26_	1210.6346	1210.6346	1255.6328	0.01	+HCOO
**21**	14.51	Ginsenoside Ra3 (Ra3)	C_59_H_100_O_27_	1240.6452	1240.6447	1285.6429	0.36	+HCOO
**22**	14.53	Ginsenoside Rb1 (Rb1)	C_54_H_92_O_23_	1108.6029	1108.6006	1153.5988	2.02	+HCOO
**23**	14.58	Gypenoside XLIX (XLIX)	C_52_H_86_O_21_	1046.5662	1046.5658	1091.564	0.31	+HCOO
**24**	15.04	Malonyl ginsenoside Rb1 (M-Rb1)	C_57_H_94_O_26_	1194.6033	1194.6057	1193.5984	2	–H
**25**	15.25	Ginsenoside Rc (Rc)	C_53_H_90_O_22_	1078.5924	1078.5925	1123.5907	0.1	+HCOO
**26**	15.47	Ginsenoside Ra1 (Ra1)	C_58_H_98_O_26_	1210.6346	1210.633	1255.6312	1.27	+HCOO
**27**	15.69	Ginsenoside Ro (Ro)	C_48_H_76_O_19_	956.4981	956.4978	955.4905	0.28	–H
**28**	15.8	Ginsenoside F1 (F1)	C_36_H_62_O_9_	638.4394	638.4395	683.4377	0.11	+HCOO
**29**	15.9	Malonyl ginsenoside Rc (M-Rc)	C_56_H_92_O_25_	1164.5928	1164.5947	1163.5874	1.6	–H
**30**	16.25	Ginsenoside Rb2 (Rb2)	C_53_H_90_O_22_	1078.5924	1078.5913	1123.5895	0.99	+HCOO
**31**	16.6	Ginsenoside Rb3 (Rb3)	C_53_H_90_O_22_	1078.5924	1078.5915	1123.5897	0.81	+HCOO
**32**	16.9	Malonyl ginsenoside Rb2 (M-Rb2)	C_56_H_92_O_25_	1164.5928	1164.5923	1163.585	0.4	–H
**33**	18.28	Gypenoside A (GyA)	C_46_H_74_O_17_	898.4926	898.4915	943.4897	1.21	+HCOO
**34**	18.29	Ginsenoside Rd (Rd)	C_48_H_82_O_18_	946.5501	946.5495	991.5477	0.59	+HCOO
**35**	18.97	Malonyl ginsenoside Rd (M-Rd)	C_51_H_84_O_21_	1032.5505	1032.5498	1031.5425	0.7	–H
**36**	20.4	Gypenoside XVII (GyXVII)	C_48_H_82_O_18_	946.5501	946.5497	991.5479	0.47	+HCOO
**37**	21.96	Notoginsenoisde Fe (nFe)	C_47_H_80_O_17_	916.5396	916.5393	961.5375	0.22	+HCOO
**38**	23.29	Compound O (CO)	C_47_H_80_O_17_	916.5396	916.5396	961.5378	0.1	+HCOO
**39**	23.46	Ginsenoside Rg4 (Rg4)	C_42_H_70_O_12_	766.4867	766.4875	811.4857	0.91	+HCOO
**40**	23.84	Ginsenoside Rk3 (Rk3)	C_36_H_60_O_8_	620.4288	620.4292	665.4274	0.51	+HCOO
**41**	24.28	Ginsenoside F4 (F4)	C_42_H_70_O_12_	766.4867	766.4865	811.4847	0.27	+HCOO
**42**	24.68	Ginsenoside Rh4 (Rh4)	C_36_H_60_O_8_	620.4288	620.4275	665.4257	2.01	+HCOO
**43**	25.15	Gypenoside L (GyL)	C_42_H_72_O_14_	800.4922	800.4905	845.4887	2.1	+HCOO
**44**	25.19	Ginsenoside F2 (F2)	C_42_H_72_O_13_	784.4973	784.4968	829.495	0.6	+HCOO
**45**	25.34	Gypenoside LI (GyLI)	C_42_H_72_O_14_	800.4922	800.4909	845.4891	1.6	+HCOO
**46**	25.57	Notoginsenoside Ft1 (nFt1)	C_47_H_80_O_17_	916.5396	916.5417	961.5399	2.26	+HCOO
**47**	25.78	Protopanaxatiol (PPT)	C_30_H_52_O_4_	476.3866	476.3862	521.3844	0.71	+HCOO
**48**	26.01	20(S)-Ginsenoside Rg3 (Rg3)	C_42_H_72_O_13_	784.4973	784.498	829.4962	0.9	+HCOO
**49**	26.2	20(R)-Ginsenoside Rg3 (Rg3-R)	C_42_H_72_O_13_	784.4973	784.4989	829.4971	1.97	+HCOO
**50**	26.37	Ginsenoisde Mc (Mc)	C_41_H_70_O_12_	754.4867	754.4867	799.4849	0	+HCOO
**51**	26.52	Compound Y (CY)	C_41_H_70_O_12_	754.4867	754.4849	799.4831	2.3	+HCOO
**52**	27.35	Compound K (CK)	C_36_H_62_O_8_	622.4445	622.4433	667.4415	1.8	+HCOO
**53**	27.43	Ginsenoside Rk1 (Rk1)	C_42_H_70_O_12_	766.4867	766.4861	811.4843	0.81	+HCOO
**54**	27.55	Ginsenoside Rg5 (Rg5)	C_42_H_70_O_12_	766.4867	766.4873	811.4855	0.73	+HCOO
**55**	27.7	Ginsenoside Rh2 (Rh2)	C_36_H_62_O_8_	622.4445	622.4441	667.4423	0.52	+HCOO
**56**	29.04	Ginsenoside Rk2 (Rk2)	C_36_H_60_O_7_	604.4339	604.4341	649.4323	0.35	+HCOO
**57**	29.15	Ginsenoside Rh3 (Rh3)	C_36_H_60_O_7_	604.4339	604.434	649.4322	0.17	+HCOO
**58**	30.04	Protopanaxadiol (PPD)	C_30_H_52_O_3_	460.3916	460.3917	505.3899	0.19	+HCOO

**Table 2:** Quantification of 39 ginsenosides from the roots, stems and leaves, and berries of *P. ginseng*. Values are expressed as mg/g.

No.	Ginsenosides	Roots	Stems and Leaves	Berries	No.	Ginsenosides	Root	Stems and Leaves	Berry
1	gRf	1.8 ± 0.1	0.3 ± 0.1	3.6 ± 1.1	21	Ro	0.15 ± 0.01	0.014 ± 0.002	0.014 ± 0.002
2	nR1	0.06 ± 0.01	0.03 ± 0.01	1.2 ± 0.2	22	M-Rc	0.9 ± 0.1	1.08 ± 0.04	4.7 ± 0.5
3	Rg1	3.1 ± 0.2	9.6 ± 0.5	8.9 ± 4.3	23	Rb2	0.6 ± 0.1	2.8 ± 0.1	2.3 ± 0.6
4	Re	3.0 ± 0.3	17 ± 1	26 ± 8	24	Rb3	not detected	0.5 ± 0.1	not detected
5	vR4	0.007 ± 0.001	1.2 ± 0.1	0.9 ± 0.1	25	M-Rb2	0.9 ± 0.1	1.6 ± 0.1	6.8 ± 0.5
6	Rf	4.6 ± 0.2	0.30 ± 0.01	3.4 ± 1.1	26	Rd	0.15 ± 0.01	7.1 ± 0.2	2.3 ± 0.5
7	nR2	1.1 ± 0.1	0.7 ± 0.1	4.5 ± 1.3	27	M-Rd	0.014 ± 0.001	3.1 ± 0.1	5.3 ± 0.4
8	nR4	0.14 ± 0.02	not detected	not detected	28	CO	0.06 ± 0.01	12.6 ± 0.1	0.4 ± 0.1
9	F5	not detected	5.8 ± 0.2	2.8 ± 0.3	29	Rg4	not detected	not detected	0.06 ± 0.03
10	Rh1	0.021 ± 0.002	0.24 ± 0.01	0.04 ± 0.02	30	F4	not detected	0.4 ± 0.1	2.1 ± 0.8
11	Rg2	0.17 ± 0.01	0.61 ± 0.03	0.6 ± 0.5	31	F2	0.0019 ± 0.0006	2.7 ± 0.3	0.03 ± 0.01
12	F3	not detected	5.3 ± 0.2	4.1 ± 0.4	32	Rg3	0.0004 ± 0.0002	0.09 ± 0.01	0.007 ± 0.001
13	Rg2-R	not detected	0.008 ± 0.001	0.06 ± 0.03	33	Mc	not detected	0.24 ± 0.04	not detected
14	F1	not detected	not detected	0.005 ± 0.001	34	CY	not detected	0.08 ± 0.02	not detected
15	Ra2	3.2 ± 0.1	not detected	not detected	35	CK	0.0010±0.0004	0.003±0.001	0.002 ± 0.001
16	Ra3	0.9 ± 0.1	not detected	not detected	36	Rk1	not detected	1.18±0.02	1.153 ± 0.004
17	Rb1	3.8 ± 0.3	0.08 ± 0.01	2.8 ± 0.6	37	Rg5	0.031 ± 0.004	not detected	0.08 ± 0.01
18	M-Rb1	4.7 ± 0.5	0.7 ± 0.1	9.9 ± 1.2	38	Rh2	not detected	0.011 ± 0.002	not detected
19	Rc	0.9 ± 0.1	3.5 ± 0.1	not detected	39	Rh1-R	not detected	0.0028 ± 0.0004	not detected
20	Ra1	0.6 ± 0.2	not detected	not detected					

## Supplementary Materials

Supplementary materials are available online. Figure S1. Molecular structures of 58 ginsenosides listed in Table 1. Table S1. Validation study for the analysis of the 58 ginsenosides using UPLC-QTOF/MS. Table S1. Pharmacological activities of 17 ginsenosides which are mainly or uniquely consisted in ginseng root (GR), stem and leaf (GS), and berry (GB).
