# Supplementary material for: Comprehensive Profiling and Quantification of Ginsenosides in the Root, Stem, Leaf, and Berry of Panax ginseng by UPLC-QTOF/MS
Source: Molecules. 2017 Dec 4;22(12):2147. doi: 10.3390/molecules22122147 (PMC6149965; doi:10.3390/molecules22122147)

Figure S1. Molecular structures of 58 ginsenosides listed in Table 1.

(1) 20-O-Glucoginsenoside Rf

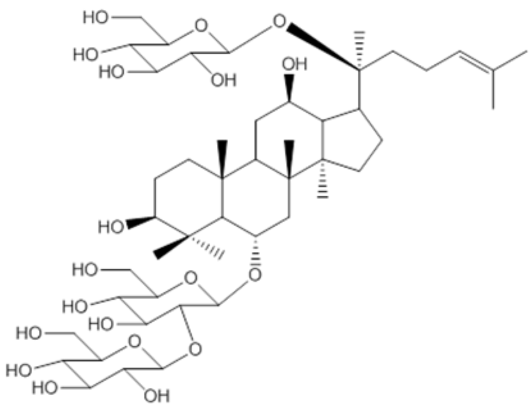

(2) Notoginsenoside R1

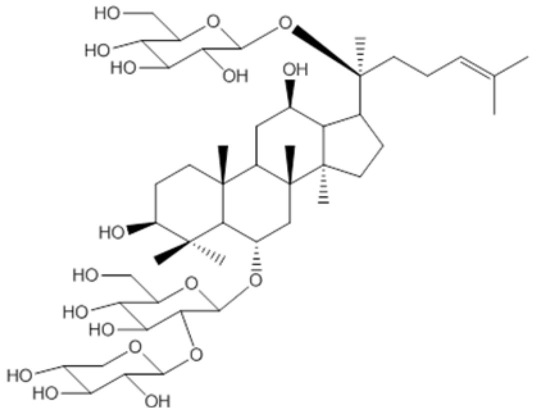

(3) Ginsenoisde Rg1

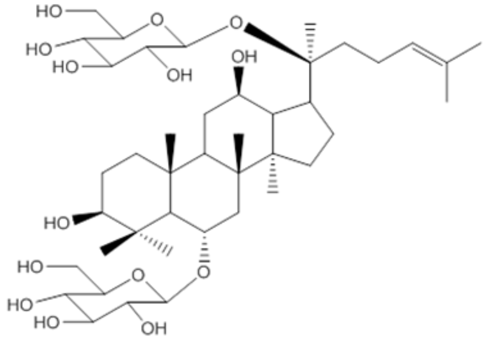

(4) Ginsenoside Re

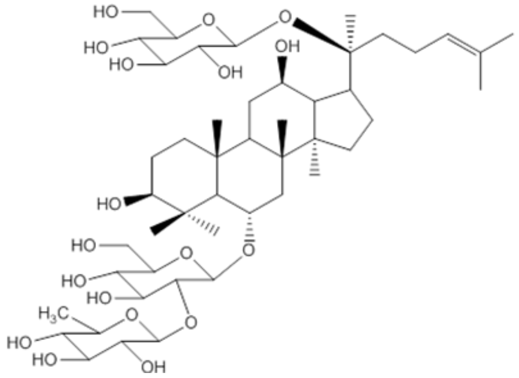

(5) Floralginsenoside Ka

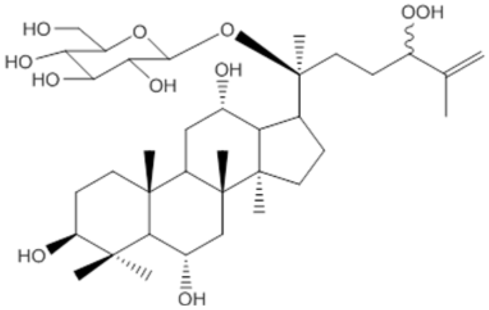

(6) Ginsenoside Rh6

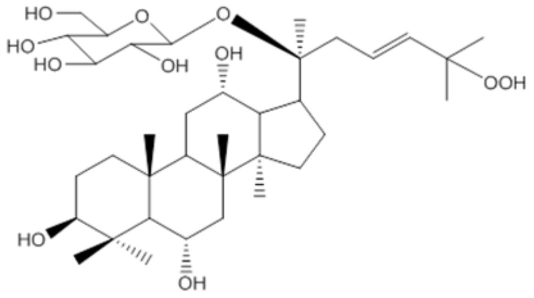

(7) Ginsenoside Rh23

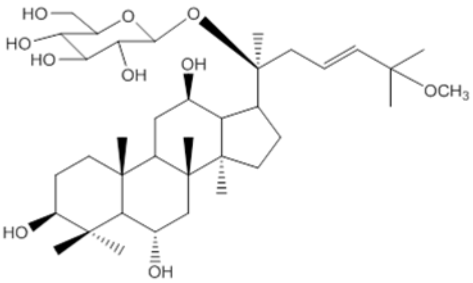

(8) Vinaginsenoside R4

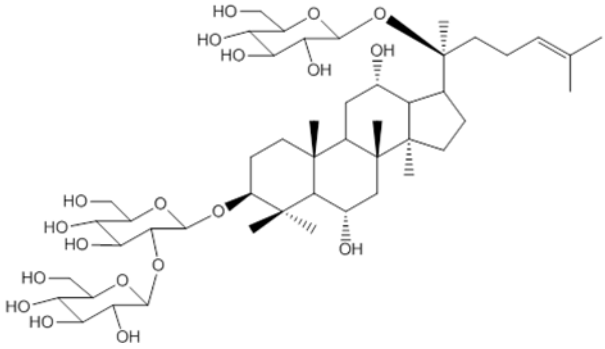

(9) Pseudo-ginsenoside F11

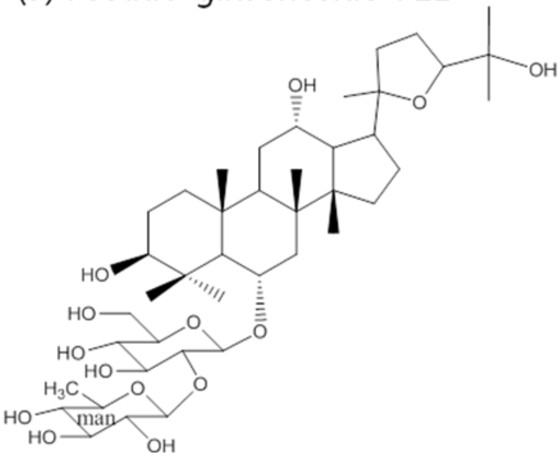

(10) Ginsenoside Rf

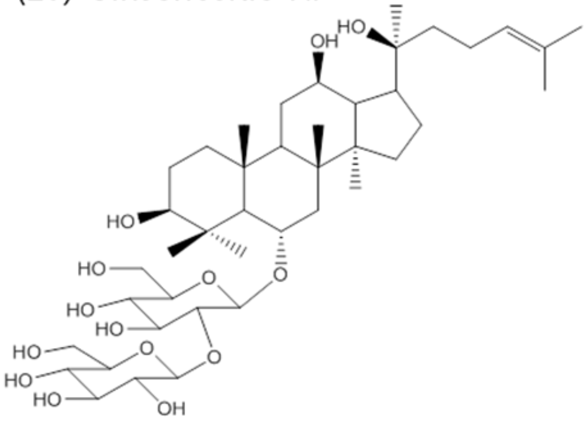

(11) Notoginsenoside R2

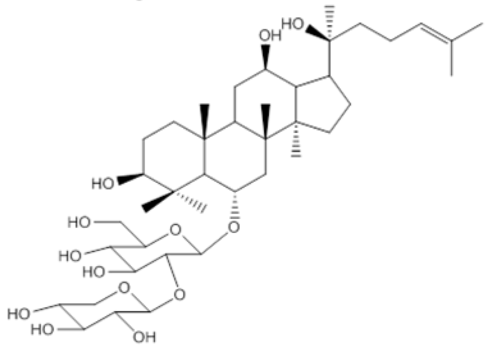

(12) Notoginsenoside R4

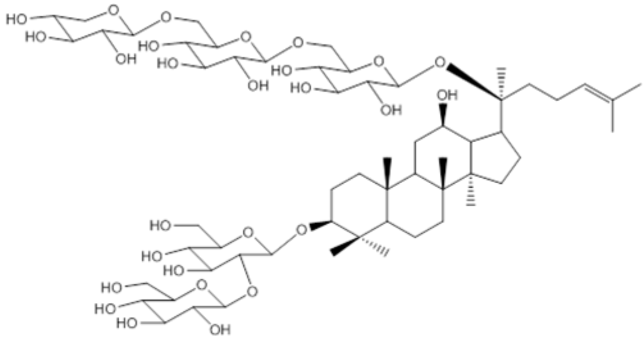

(13) Ginsenoside F5

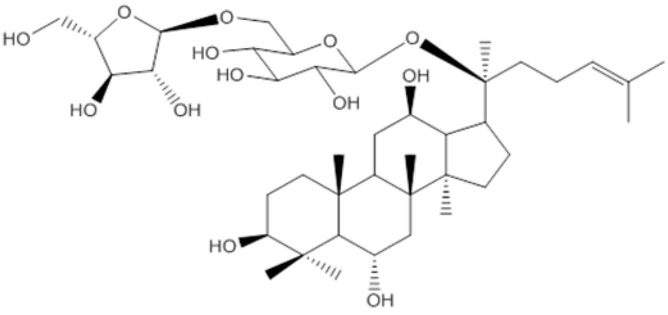

(14) Ginsenoside Rh1

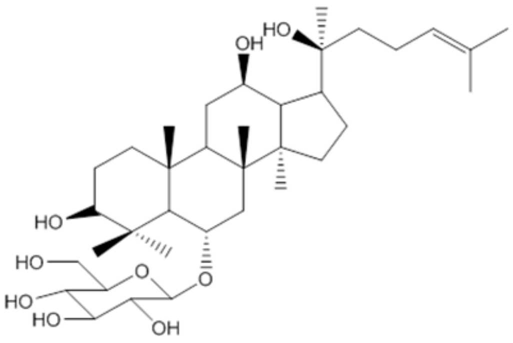

(15) 20(R)-Notoginsenoside R2

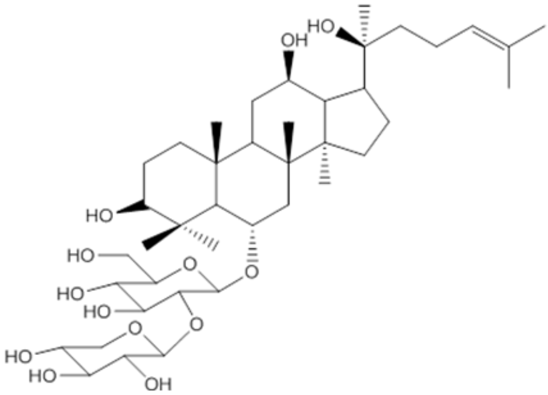

(16) Ginsenoside Rg2

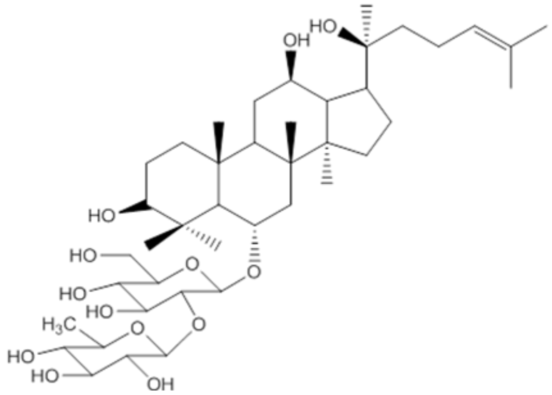

(17) Ginsenoside F3

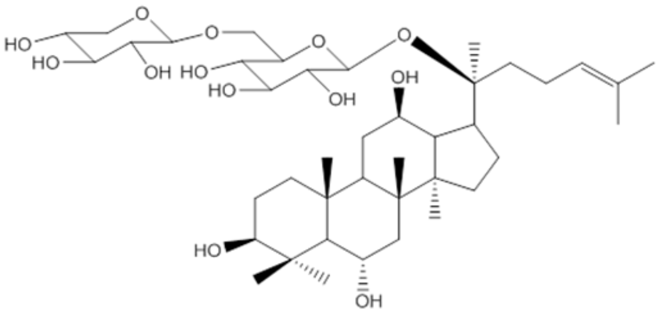

(18) 20(R)-Ginsenoside Rg2

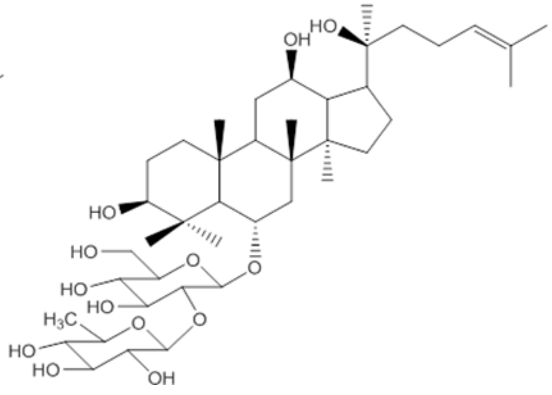

(19) 20(R)-Ginsenoside Rh1

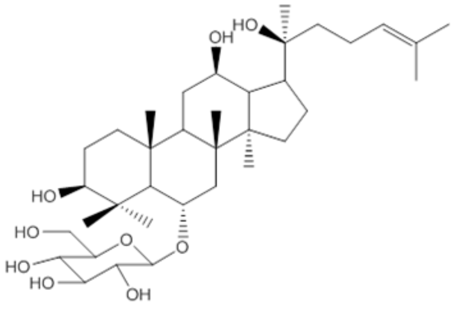

(20) Ginsenoside Ra2

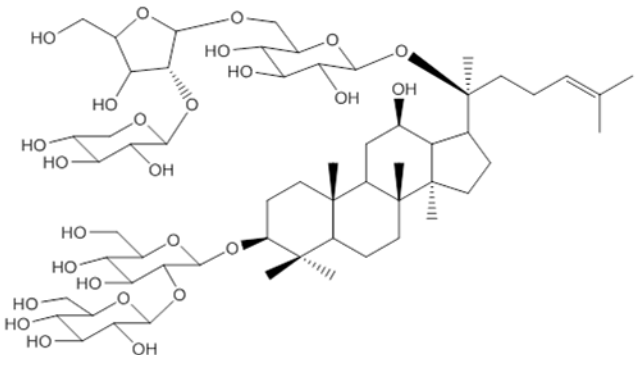

(21) Ginsenoside Ra3

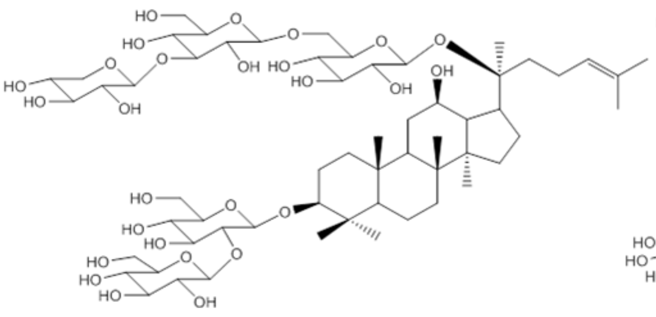

(22) Ginsenoside Rb1

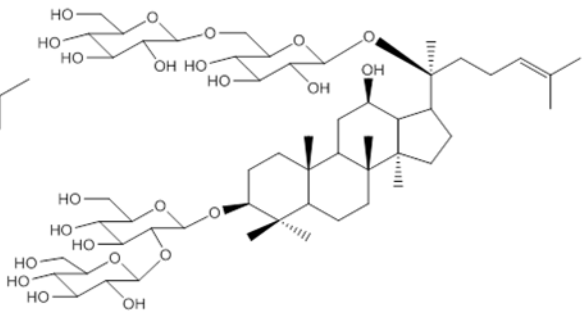

(23) Gypenoside XLIX

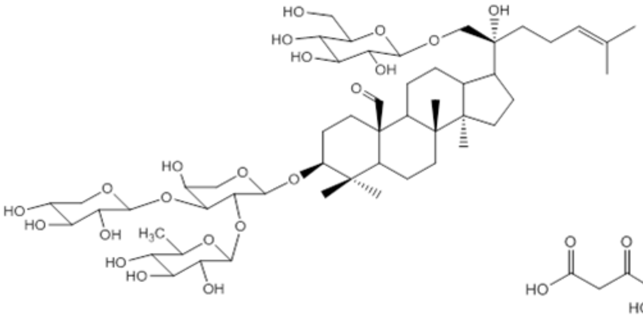

(24) Malonyl ginsenoside Rb1

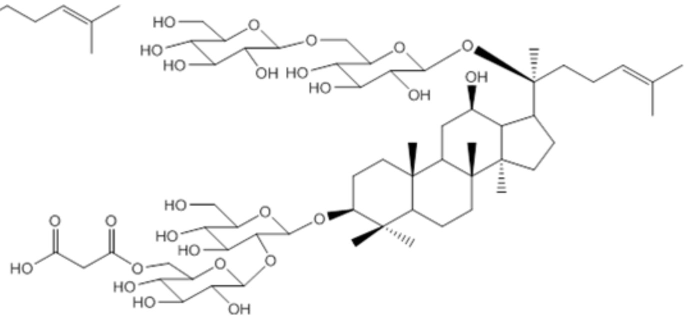

(25) Ginsenoside Rc

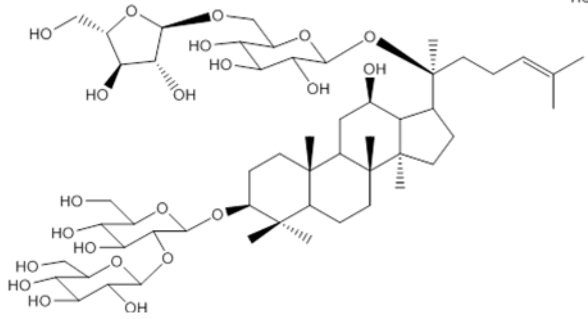

(26) Ginsenoside Ra1

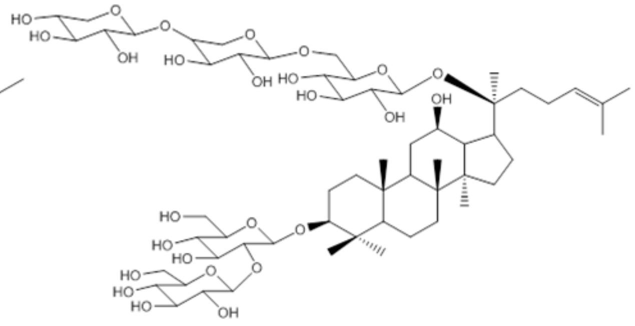

(27) Ginsenoside Ro

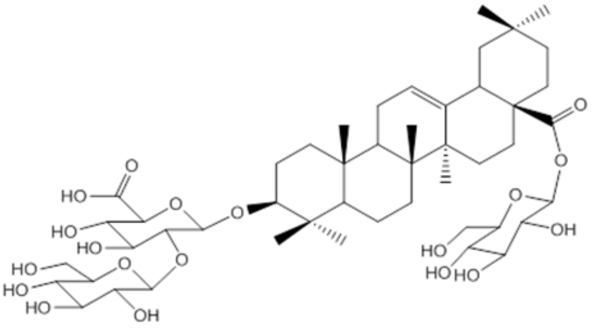

(28) Ginsenoside F1

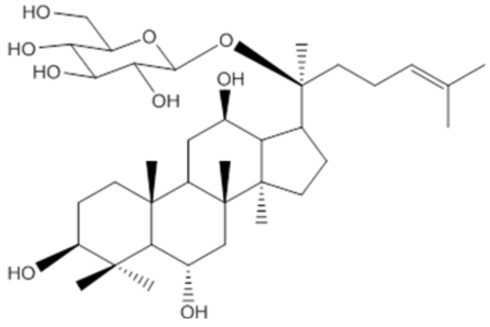

(29) Malonyl ginsenoside Rc

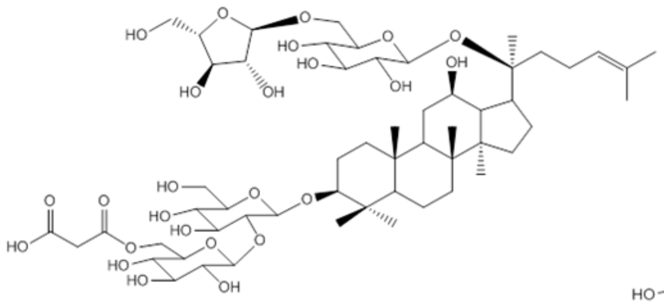

(30) Ginsenoside Rb2

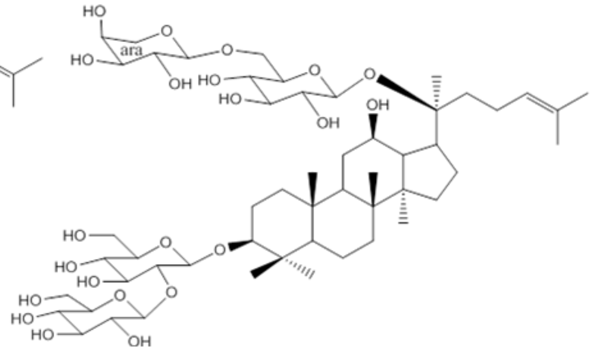

(31) Ginsenoside Rb3

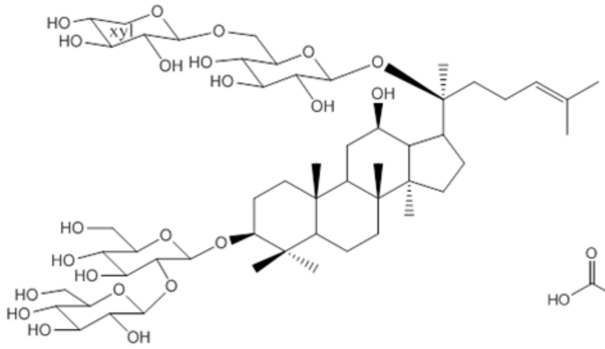

(32) Malonyl ginsenoside Rb2

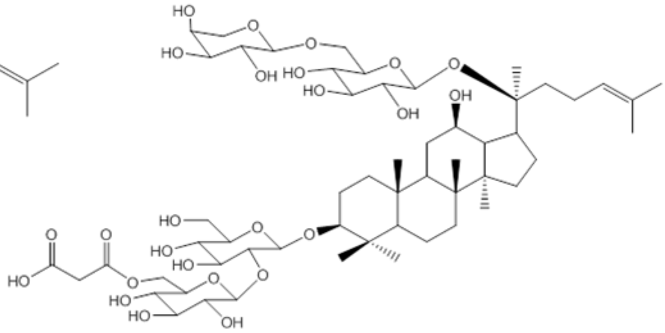

(33) Gypenoside A

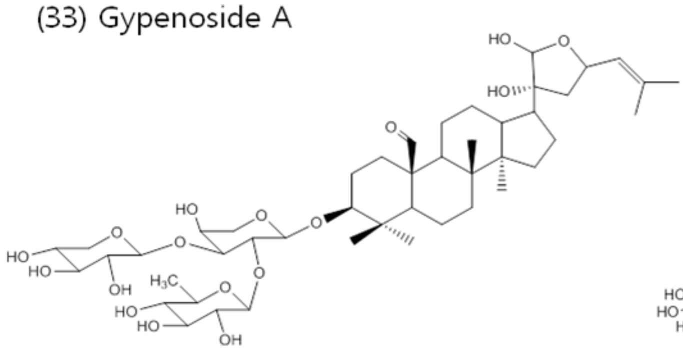

(34) Ginsenoside Rd

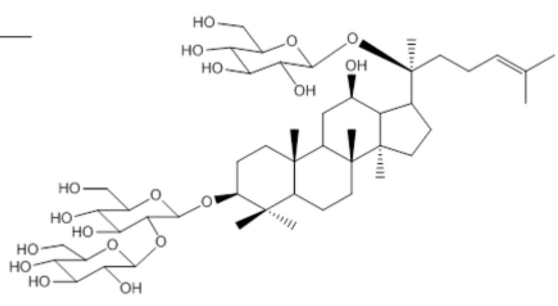

(35) Malonyl ginsenoside Rd

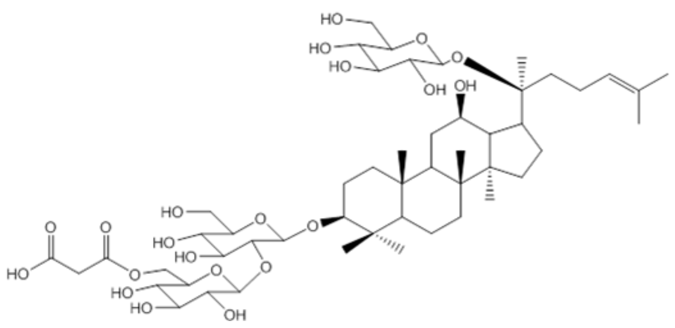

(36) Gypenoside XVII

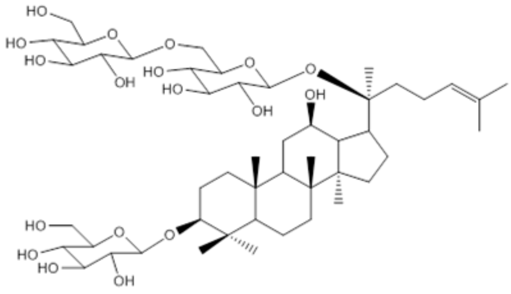

(37) Notoginsenoisde Fe

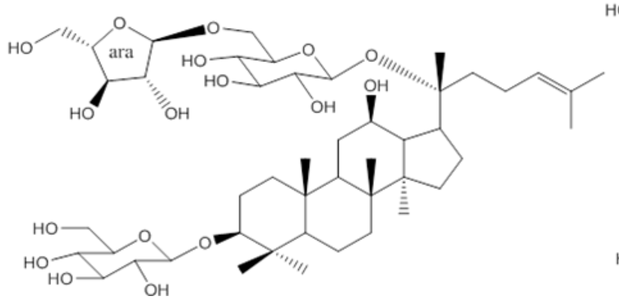

(38) Compound O

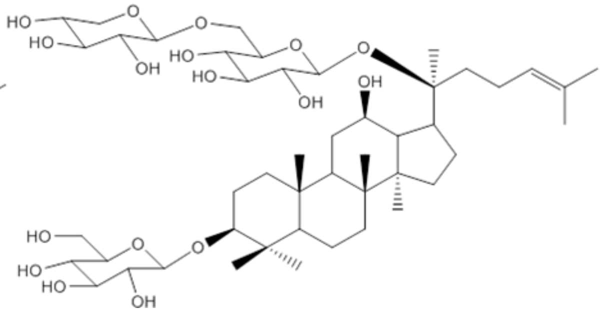

(39) Ginsenoside Rg4

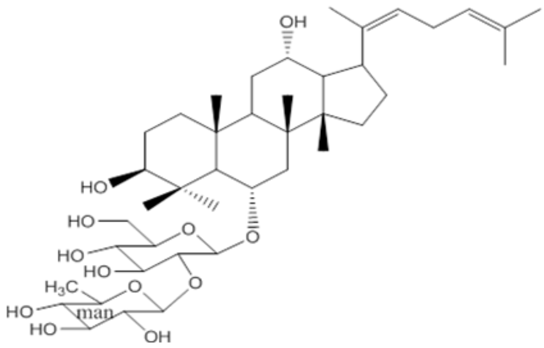

(40) Ginsenoside Rk3

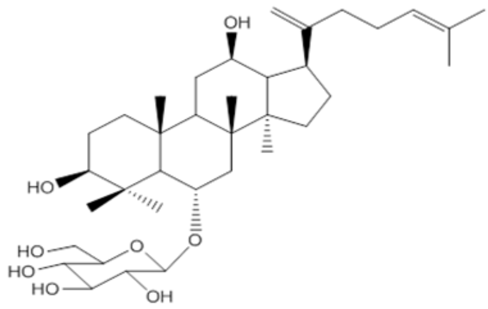

(41) Ginsenoside F4

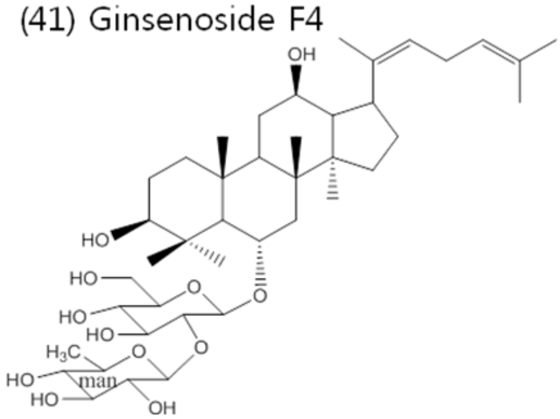

(42) Ginsenoside Rh4

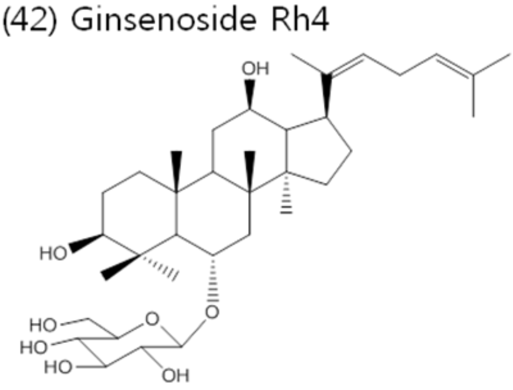

(43) Gypenoside L

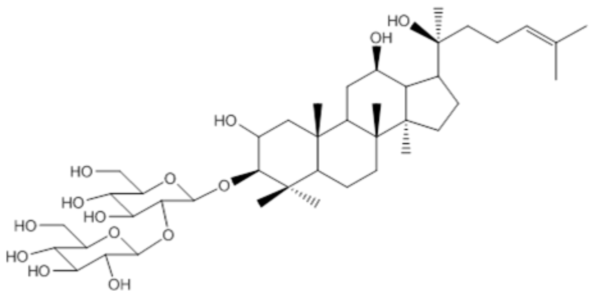

(44) Ginsenoside F2

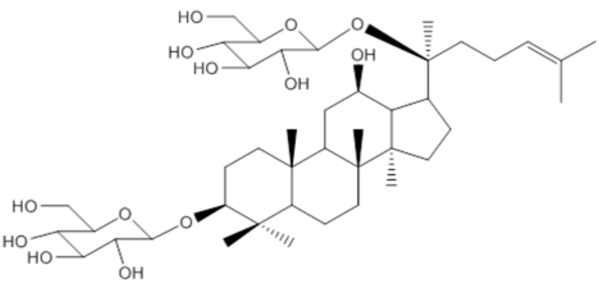

(45) Gypenoside LI

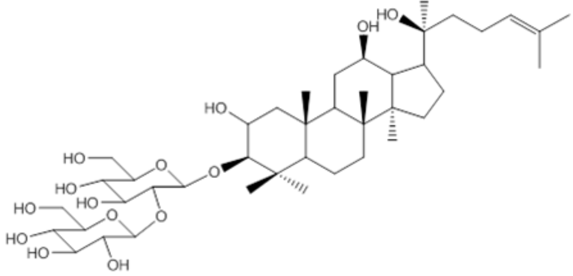

(46) Notoginsenoside Ft1

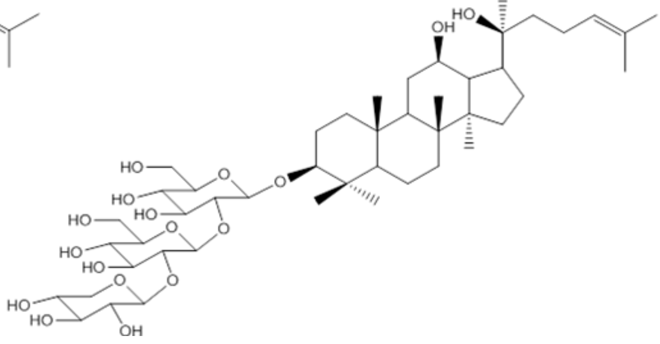

(47) Protopanaxatiol

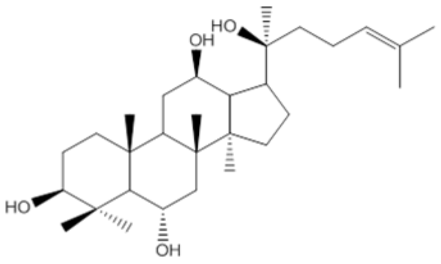

(48) 20(S)-Ginsenoside Rg3

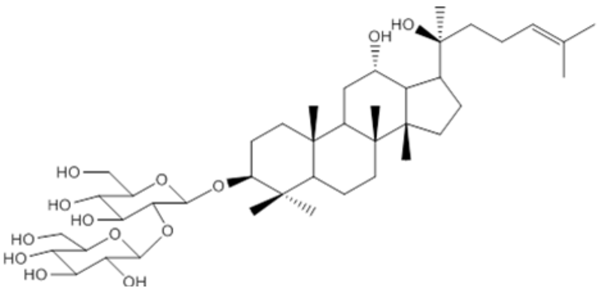

(49) 20(R)-Ginsenoside Rg3

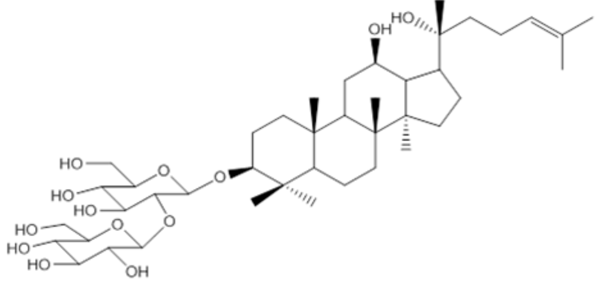

(50) Ginsenoisde Mc

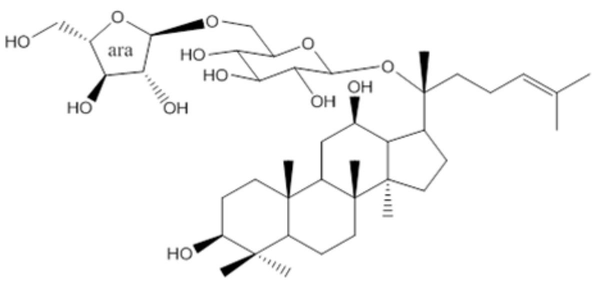

(51) Compound Y

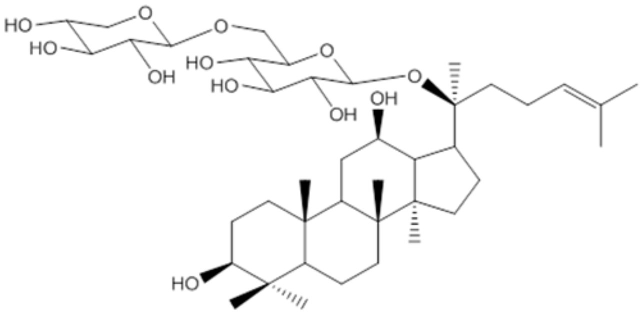

(52) Compound K

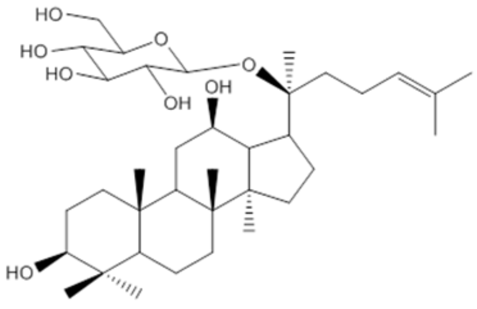

(53) Ginsenoside Rk1

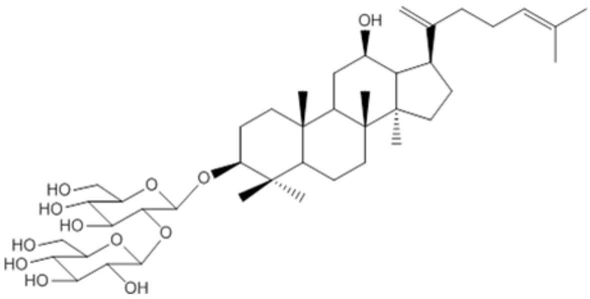

(54) Ginsenoside Rg5

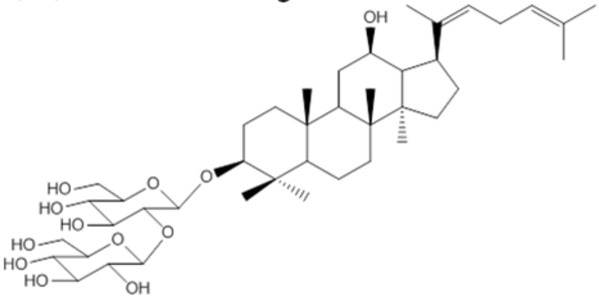

(55) Ginsenoside Rg5

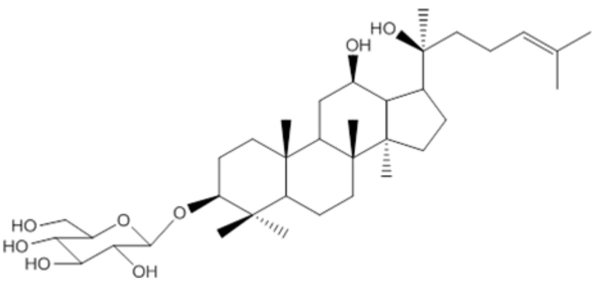

(56) Ginsenoside Rk2

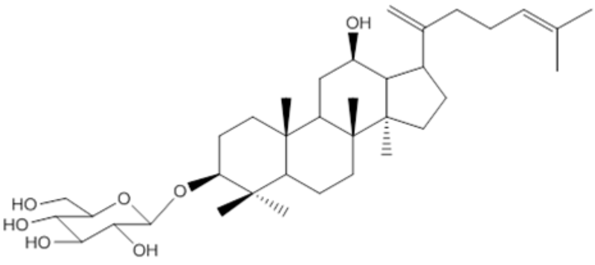

(57) Ginsenoside Rk2

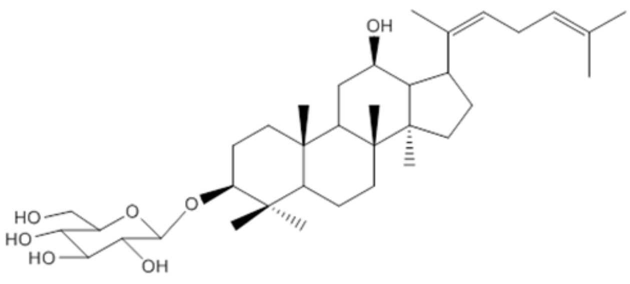

(58) Protopanaxadiol

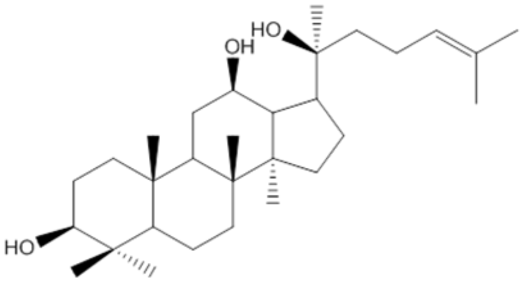

Supplement: Supplementary file 1 [file molecules-22-02147-s001.zip › Supplemental figure 1.pdf]
