# Supplementary material for: Comprehensive Profiling and Quantification of Ginsenosides in the Root, Stem, Leaf, and Berry of Panax ginseng by UPLC-QTOF/MS
Source: Molecules. 2017 Dec 4;22(12):2147. doi: 10.3390/molecules22122147 (PMC6149965; doi:10.3390/molecules22122147)
Supplement: Supplementary file 1 [file molecules-22-02147-s001.zip › Supplemental table 1.doc]

**Table S1.** Validation study for the analysis of 58 ginsenosides using UPLC-QTOF/MS

| No. | Ginsenosides | Correlation (*R^2^*) | Linear range (pg) * | LOD (pg) | No. | Ginsenosides | Correlation (*R^2^*) | Linear range (pg) | LOD (pg) |
| --- | --- | --- | --- | --- | --- | --- | --- | --- | --- |
| 1 | gRf | 0.9908 | 200 – 20000 | 132 | 30 | Rb2 | 0.9960 | 20 – 40000 | 4 |
| 2 | nR1 | 0.9959 | 020 – 20000 | 18 | 31 | Rb3 | 0.9954 | 20 – 20000 | 8 |
| 3 | Rg1 | 0.9985 | 50 – 40000 | 24 | 32 | M-Rb2 | 0.9939 | 20 – 40000 | 2 |
| 4 | Re | 0.9941 | 20 – 120000 | 6 | 33 | GyA | 0.9989 | 50 – 20000 | 10 |
| 5 | fKa | 0.9955 | 50 – 20000 | 24 | 34 | Rd | 0.9924 | 20 – 40000 | 2 |
| 6 | Rh6 | 0.9935 | 50 – 4000 | 16 | 35 | M-Rd | 0.9926 | 20 – 40000 | 4 |
| 7 | Rh23 | 0.9960 | 20 – 1600 | 4 | 36 | GyXVII | 0.9987 | 20 – 2000 | 6 |
| 8 | vR4 | 0.9992 | 50 – 1000 | 10 | 37 | Fe | 0.9988 | 50 – 2000 | 30 |
| 9 | pF11 | 0.9963 | 20 – 20000 | 8 | 38 | CO | 0.9915 | 200 – 20000 | 110 |
| 10 | Rf | 0.9935 | 50 – 24000 | 16 | 39 | Rg4 | 0.9999 | 50 – 10000 | 34 |
| 11 | nR2 | 0.9972 | 100 – 40000 | 74 | 40 | Rk3 | 0.9951 | 100 – 20000 | 58 |
| 12 | nR4 | 0.9979 | 50 – 8000 | 24 | 41 | F4 | 0.9942 | 500 – 20000 | 360 |
| 13 | F5 | 0.9963 | 20 – 20000 | 4 | 42 | Rh4 | 0.9969 | 200 – 80000 | 106 |
| 14 | Rh1 | 0.9966 | 50 – 20000 | 10 | 43 | GyL | 0.9991 | 20 – 5000 | 2 |
| 15 | R2-R | 0.9911 | 20 – 320 | 6 | 44 | F2 | 0.9980 | 20 – 3800 | 6 |
| 16 | Rg2 | 0.9970 | 20 – 3200 | 8 | 45 | GyLI | 0.9994 | 20 – 5000 | 2 |
| 17 | F3 | 0.9988 | 50 – 20000 | 12 | 46 | nFt1 | 0.9925 | 20 – 20000 | 4 |
| 18 | Rg2-R | 0.9986 | 50 – 4000 | 12 | 47 | PPT | 0.9934 | 200 – 20000 | 134 |
| 19 | Rh1-R | 0.9984 | 50 – 20000 | 12 | 48 | Rg3 | 0.9987 | 20 – 6000 | 2 |
| 20 | Ra2 | 0.9981 | 500 – 72000 | 300 | 49 | Rg3-R | 0.9993 | 50 – 5600 | 10 |
| 21 | Ra3 | 0.9978 | 20 – 30000 | 2 | 50 | Mc | 0.9952 | 20 – 20000 | 2 |
| 22 | Rb1 | 0.9981 | 20 – 74000 | 6 | 51 | CY | 0.9982 | 20 – 20000 | 2 |
| 23 | GyXLIX | 0.9977 | 50 – 20000 | 32 | 52 | CK | 0.9983 | 20 – 1800 | 8 |
| 24 | M-Rb1 | 0.9927 | 50 – 100000 | 14 | 53 | Rk1 | 0.9973 | 500 – 150000 | 256 |
| 25 | Rc | 0.9971 | 50 – 40000 | 24 | 54 | Rg5 | 0.9988 | 200 – 20000 | 80 |
| 26 | Ra1 | 0.9967 | 100 – 80000 | 54 | 55 | Rh2 | 0.9964 | 20 – 10000 | 8 |
| 27 | Ro | 0.9985 | 20 – 8000 | 2 | 56 | Rk2 | 0.9948 | 50 – 20000 | 22 |
| 28 | F1 | 0.9985 | 50 – 20000 | 15 | 57 | Rh3 | 0.9996 | 50 – 1000 | 32 |
| 29 | M-Rc | 0.9976 | 20 – 38000 | 6 | 58 | PPD | 0.9969 | 500 – 20000 | 158 |

* On-column amounts; LOD: Limit of detection
