# Supplementary material for: Comprehensive Profiling and Quantification of Ginsenosides in the Root, Stem, Leaf, and Berry of Panax ginseng by UPLC-QTOF/MS
Source: Molecules. 2017 Dec 4;22(12):2147. doi: 10.3390/molecules22122147 (PMC6149965; doi:10.3390/molecules22122147)
Supplement: Supplementary file 1 [file molecules-22-02147-s001.zip › Supplemental table 2.doc]

**Table S2.** Pharmacological activities of 17 ginsenosides which are mainly or uniquely consisted in ginseng root (GR), stem and leaf (GS), and berry (GB)

| Ginseng parts | Character | Ginsenosides | Pharmacological activities | | | | | |
| --- | --- | --- | --- | --- | --- | --- | --- | --- |
|  |  |  | Anti  -cancer | Anti  -diabetes | Anti  -inflammation | Lymphocyte proliferative capacity | Immunomodulating activity | Chemopreventive effect |
| GR | Main^1)^ | Rf | - | - | O [43] | - | - | - |
|  |  | Rb1 | - | - | - | O [46] | - | - |
|  |  | Ro | - | - | - | O [46] | - | - |
|  | Unique^2)^ | - | - | - | - | - | - | - |
| GS | Main | Rg1 | - | - | O [5] | - | O [5] | - |
|  |  | Rc | O [6]^3)^ | - | - | O [46] | - | - |
|  |  | Rb2 | O [6] | O [42] | O [44] | O [46] | - | O [15] |
|  |  | Rd | - | O [42] | O [44] | - | - | - |
|  |  | F2 | O [38] | - | - | - | - | - |
|  |  | Rg3 | O [6, 39] | - | - | - | - | - |
|  |  | CK | O [6] | - | - | - | - | - |
|  | Unique | Rb3 | O [39] | - | - | - | - | O [15] |
|  |  | Rh2 | O [6,39] | - | - | - | - | - |
|  |  | Rh1-R | O [40] | - | O [45] | - | - | - |
| GB | Main | Re | - | O [42] | O [44] | O [46] | - | - |
|  |  | Rg2 | O [39] | - | O [44] | - | - | - |
|  |  | Rg2-R | O [39] | - | - | - | - | - |
|  | Unique | F1 | O [41] | - | - | - | - | - |

1) Main: Ginsenosides which are mainly consisted in each ginseng part

2) Unique: Ginsenosides which are uniquely consisted in each ginseng part

3) Reference
